# Impact of *in utero* drug exposure on neonates requiring ECMO: A retrospective cohort study

**DOI:** 10.3389/fped.2023.1020716

**Published:** 2023-03-27

**Authors:** Hallie Walther, Aric Schadler, Karen Garlitz, John A. Bauer, Lindsay Kohler, Erika Waldsmith, Hubert O. Ballard

**Affiliations:** ^1^ College of Medicine, University of Kentucky, Lexington, KY, United States; ^2^ Department of Pediatrics, Kentucky Children's Hospital, College of Medicine, University of Kentucky, Lexington, KY, United States; ^3^ College of Pharmacy, University of Kentucky, Lexington, KY, United States

**Keywords:** neonatal abstinence syndrome, extracorporeal membrane oxygenation (ECMO), neonate, *in utero* drug exposure, retrospective cohort analysis

## Abstract

The incidence of *in utero* drug exposure (IUDE) and neonatal extracorporeal membrane oxygenation (ECMO) utilization have both increased over the past decade. However, there are no studies to date that examine the impact that IUDE has on neonates requiring ECMO. In this retrospective cohort study, we compared the clinic course and outcomes of neonates who were placed on ECMO with IUDE vs. neonates without IUDE. Analysis included data extracted from medical records from all neonatal ECMO runs between January 2014 and January 2021 at the University of Kentucky Children's Hospital. A total of 56 neonatal patients were placed on ECMO during this time period and there were a total of 57 ECMO runs. Nearly one-third of neonates (16) had documented IUDE. There were no differences in gestational age, length of ECMO run, survival to discharge, or number of major complications while on ECMO in the neonates with IUDE compared to those without. In contrast, greater use of sedative and analgesic adjuvant medications during ECMO was required for IUDE-ECMO cases (*p *< 0.01). Trending results indicated that post-ECMO feeding complications and total hospitalization length were also greater in the IUDE-ECMO group. These findings illustrate the complex influence of prenatal drug exposures on neonatal patient care and warrant the development of clinical care strategies optimized for this unique patient group.

## Introduction

Extracorporeal membrane oxygenation (ECMO) is an advanced life-support modality used for the treatment of respiratory and cardiac failure in critically ill neonates who are not responsive to conventional therapies. The use of ECMO in neonates has increased over the past decade and was utilized 6,656 times in this patient group between 2015 and 2020 in the United States (1). Clinical outcomes for neonatal ECMO can be excellent and are often substantially better than older age pediatric patients or adults (1). A critical clinical component of successful neonatal ECMO therapy involves monitoring and maintaining a proper level of patient comfort and sedation. This typically requires continuous infusions of one or more sedative and analgesic medications (2–4) and continuous monitoring of patient status. Sedation of neonates on ECMO is complicated by numerous factors, including the pharmacokinetic variability related to gestational age and the relative circuit volume, the sequestration of drugs in the ECMO circuit, the development of tolerance to sedative medications, and ECMO-related physiologic and metabolic alterations (5–16). The duration and severity of diseases in neonates requiring ECMO often requires a prolonged course and high doses of sedative and analgesic drugs, as well as nearly continuous assessment of sedation status and dose adjustments (3, 4, 8, 10).

A recently emerging challenge in the sedation of neonates on ECMO is related to the increasing incidence of intrauterine drug exposure (IUDE). In recent years, IUDE has risen dramatically, corresponding with the rise of the opioid epidemic (17–19). This has been especially true for the region our institution serves (the state of Kentucky and central Appalachian region of the United States). A national survey of neonatal intensive care units (NICUs) found that IUDE leading to neonatal abstinence syndrome (NAS) accounted for 4% of all NICU hospital days nationwide, with some centers reporting that over 20% of NICU days were attributed to the care of infants with NAS (17). It is, therefore, likely that the frequency of infants with IUDE who require ECMO has also increased. The impact of IUDE in neonates who are critically ill is not well-documented, but this is a likely factor complicating their hospital course. Despite the increasing number of neonates with IUDE, and the importance of sedation management in neonatal ECMO, there have been no reports describing the impact of prenatal drug exposures in this special clinical setting.

Sedation management in neonates on ECMO is challenging in all infants but is further complicated in the setting of IUDE. Exposure to drugs *in utero* can lead to tolerance to sedative medications routinely used in the NICU (20). Additionally, the withdrawal symptoms that patients with IUDE experience may necessitate increased doses of these medications to maintain neonates’ comfort. To our knowledge, no studies have examined the use of sedatives in this population. Adequate sedation is essential during neonatal ECMO to avoid pain and discomfort, but oversedation and prolonged duration of sedation will make the post-ECMO course more complicated (2, 10, 21, 22). Therefore, it is crucial to gain a better understanding of how to maintain sedation goals in this population.

In this study, we sought to characterize the clinical course of neonatal patients with documented IUDE who require ECMO focusing on (1) patient outcomes, (2) sedation requirements, and (3) nutritional requirements. Comparisons were made to ECMO patients from the same institution and timeframe who did not have IUDE.

## Methods

### Participants

We performed a retrospective chart review of all neonatal patients placed on ECMO between January 2014 and January 2021 at the University of Kentucky Children's Hospital. A total of 56 neonates were identified; one patient was placed on ECMO twice, resulting in a total of 57 ECMO runs. No patients who received neonatal ECMO during this time period were excluded from the study. Approval for this study was obtained through the University of Kentucky Institutional Review Board (IRB).

### Study design

This study was designed as a retrospective cohort. Using data extracted from medical records, we compared the clinical course of neonates that had IUDE prior to ECMO requirement vs. those only requiring ECMO at our institution. Cases involving IUDE were identified by one or more of the following: an abnormal urine drug screen during the last trimester of pregnancy identified *via* maternal medical record, enrollment of the mother in an institutional prenatal medication-assisted treatment (MAT) program, or description of drug exposure in the neonatal delivery note and/or NICU patient medical record. Due to the severity of illness and degree of patient instrumentation, we were not practically able to use clinical scoring assessments to identify NAS. Data from each neonate were analyzed for birth weight, gestational age, mode of delivery, sex, diagnosis, complications during ECMO, duration of ECMO, survival to discharge, length of stay, time until full feeds, and sedation requirements. Data on sedation included medication type, number of medications, and dosage. Total oral morphine equivalents (OME) were calculated in order to standardize the dosing comparison of the various narcotics that were utilized among patients (23). ECMO complications were reported based on ESLO guidelines. Time until full P.O. feeds was calculated by determining the date where the neonate took 100% of their feeds by mouth. If an infant received a G-tube, their total length of stay was used as their time to full P.O. feeds.

### Sedation protocol

Induction and maintenance of sedation in all neonatal ECMO cases were performed identically using institutional standard clinical practice guidelines. Per NICU protocol, depth of sedation was determined hourly using the Richmond Agitation-Sedation Scale (RASS) (24) and adequate sedation was defined as a RASS score of 0 to −2 with the patient being awake, but not agitated or uncomfortable; patient status was verified hourly and dose adjustments were determined by the bedside team (including a physician, a pharmacist, and nursing specialists). During cannulation, neonates were given bolus injections of fentanyl and midazolam. Following cannulation, patients were started on a morphine drip at 10–20 μg/kg/h and midazolam drip at 0.1 mg/kg/h. Fentanyl, dexmedetomidine, phenobarbital, lorazepam, diazepam, hydromorphone, clonidine, and ketamine were each available as analgesic adjuvants. Methadone and buprenorphine were available for the treatment of withdrawal. Once stabilized on ECMO, patients underwent daily sedation holidays to prevent the development of tolerance to sedative and analgesic medications. Following sedation holidays, drips were restarted at 10% less than their prior dose. The use of paralytic agents was minimized in order to allow for hourly neurological examination.

### Statistical methods

Initial review of the collected data set showed that nearly all variables were skewed and non-normally distributed. For these reasons, we used nonparametric statistical testing between groups. Descriptive statistics were reported as median (interquartile range) for continuous variables and count (percentage) for categorical data. Categorical data about the demographic and clinical characteristics were analyzed using Pearson's chi-squared or Fisher's exact test as appropriate. Continuous variables were analyzed utilizing nonparametric methods with independent-samples difference of medians test. An alpha level of 0.05 was used to identify significance. All statistical analysis was performed using IBM SPSS Statistics version 28.

## Results

### ECMO patient population and baseline characteristics

Table 1 identifies the patient characteristics for neonates who received ECMO following IUDE vs. those without IUDE. During the period studied, approximately one-third of the neonatal ECMO cases at our institution had IUDE (16 of 56, 28%). The most common conditions indicating the need for ECMO in both groups were persistent pulmonary hypertension, shock, and meconium aspiration syndrome. Neonates with IUDE were more likely to have meconium aspiration syndrome than neonates without IUDE (43.8% vs. 17.5%, *p *= 0.04). The majority of neonates with and without IUDE were placed on veno-venous (VV) ECMO. Gestational age and frequency of vaginal birth were not different between groups. The birth weight of the neonates in the IUDE group tended to be lower than those without IUDE (2,868.5 g vs. 3,314.5 g, *p *= 0.14), with a greater fraction of patients less than 2 kg in the IUDE group. There was also no difference in total ECMO run time between groups.

**Table 1:** Characteristics of neonates requiring ECMO.

	ECMO only control (*n* = 40)	IUDE-ECMO (*n* = 16)	*p*-value
**Patient characteristics**			
Gestational age (weeks), median (IQR)	38.1 (36.6–40.0)	38.2 (35.9–39.2)	0.90
Birth weight (g), median (IQR)	3,314 (2767–3686)	2,868 (2308–3450)	0.14
Vaginal delivery, *n* (%)	20 (50)	8 (50)	1.00
**Indication(s) for ECMO, *n* (%)**			
PPHN	36 (92)	16 (100)	0.25
Shock	5 (31)	14 (35)	0.79
Meconium aspiration	7 (17)	7 (43)	0.04
ECMO type, *n* (%)			
VA	14 (35)	6 (37)	0.86
VV	26 (65)	10 (62)	0.82
**Time to cannulation, median (IQR)**			
Hours to cannulation	50.8 (32.2–85.6)	51.5 (23.9–79.3)	0.47
**ECMO procedure time**			
Total hours on ECMO	117.1 (85.5–150.3)	89.1 (78.89–164.0)	0.38

ECMO, extracorporeal membrane oxygenation; IQR, interquartile range; IUDE, *in utero* drug exposure; MAS, meconium aspiration syndrome; P.O., by mouth; PPHN, persistent pulmonary hypertension of the newborn; VA, veno-arterial; VV, veno-venous.

### Sedation management during ECMO

Table 2 shows the sedation and analgesic dosing requirements for the two patient groups during their ECMO runs. Despite the use of an identical standard clinical protocol for sedation management, several differences were observed between groups. Neonates with IUDE required a median of five adjuvant sedative and/or analgesic medications and neonates without IUDE required a median of three adjuvants (*p *< 0.01). IUDE in ECMO patients was associated with a more than three-fold median total dose of oral morphine equivalents over the course of their ECMO run compared to neonates without IUDE (246 vs. 77.1 mg/kg), although this was marginally significant. When the total morphine equivalents used for each patient were normalized to the actual ECMO run time, there was a striking difference between groups: IUDE cases required two-fold greater morphine equivalents per hour of ECMO (0.7 vs. 1.49 mg/kg/h, *p* < 0.01).

**Table 2:** Sedation and analgesic requirements during ECMO.

	ECMO only control (*n* = 40)	IUDE-ECMO (*n* = 16)	*p*-value
	Median (IQR)	Median (IQR)	* *
**Agent and dose requirement (mg/kg)**			
Morphine			
Drip	19.9 (11.9–36.4)	34.4 (15.0–100.5)	0.14
Bolus	3.5 (2.2–8.5)	7.0 (3.0–13.8)	0.38
Oral	0.5 (0–6.6)	0 (0–8.4)	0.77
**Fentanyl**			
Drip	0 (0–0)	0 (0–0)	0.68
Bolus	10.7 (0.0–25.0)	23.5 (2.5–51.4)	0.14
**Hydromorphone drip**			
Drip	0 (0–0)	0 (0–0)	0.03
Bolus	0 (0–0)	0 (0–0)	0.14
Methadone	0 (0–0)	0 (0–8.0)	0.01
Diazepam	0 (0–0)	0 (0–13.5)	0.08
**Midazolam**			
Drip	8.9 (0.6–24.0)	15.0 (0.7–77.0)	0.77
Bolus	3.7 (1.2–6.3)	4.9 (1.5–13.0)	0.77
Phenobarbital bolus	0 (0–1.0)	0 (0–2.3)	0.89
Dexmedetomidine drip	0 (0–8.9)	49.5 (0–170.5)	0.08
**Composite measures**			
Total oral morphine equivalents	77.1 (47.2–167.0)	245.7 (89.7–639.3)	0.14
Total no. of adjuvants	3 (2–4)	5 (3.25–7.7)	<0.01
Total morphine equivalents per hour of ECMO runtime	0.7 (0.4–1.3)	1.49 (0.7–4.2)	<0.01

ECMO, extracorporeal membrane oxygenation; IQR, interquartile range; IUDE, *in utero* drug exposure.

### Clinical outcomes following ECMO

Table 3 shows comparisons of clinical outcomes following ECMO for the two groups (IUDE vs. no IUDE). No difference was seen in survival to discharge in the neonates with IUDE vs. those without IUDE (75.0% vs. 90.0%, *p *= 0.18). Neonates with IUDE required the same amount of time on oxygen (28.0 vs. 20.0, *p *= 0.49) and ventilatory support (17.0 vs. 14.5, *p *= 0.38) than neonates without IUDE. However, trending results show that neonates with IUDE did require a longer length of stay than those without IUDE (41.0 vs. 31.5 days, *p *= 0.10).

**Table 3:** Clinical outcomes following ECMO.

	ECMO only control (*n* = 40)	IUDE-ECMO (*n* = 16)	*p*-value
**Clinical outcome, *n* (%)**			
Survival to discharge	36 (90.0)	12 (75.0)	0.21
**ECMO complications**			
Intracranial hemorrhage	9 (22)	2 (12)	0.48
Sepsis	3 (7)	1 (6)	1.00
Days on oxygen, median (IQR)	20 (14.3–30.5)	28 (14.0–57.0)	0.49
Days on ventilator, median (IQR)	14.5 (11.0–19.0)	17 (12.0–28.75)	0.38
Length of stay, median (IQR)	31.5 (22.3–48.8)	41 (26.3–74.5)	0.10
**Post-ECMO feeding complications**			
Days until full P.O. feeds, median (IQR)^a^	19 (14.0–42.0)	40 (14.5–84.3)	0.24
Full P.O. feeds by discharge, *n* (%)^a^	25 (69.4)	7 (58.3)	0.48
Gastric tube, *n* (%)^a^	6 (16.7)	3 (25.0)	0.67

ECMO, extracorporeal membrane oxygenation; IQR, interquartile range; IUDE, *in utero* drug exposure.

^a^ Only infants who survived until discharge were included in this analysis.

### Nutrition

There was no difference observed in the percentage of neonates with and without IUDE who reached full P.O. feeds by the time of discharge (69.4% vs. 58.3%, *p *= 0.48) (Table 2). Of these infants, it took a median length of 40 days for neonates with IUDE to reach full P.O. feeds compared to 19 days in neonates without IUDE (*p *= 0.24) (Table 3). There was also no difference seen in the amount who required a G-tube (25.0% vs. 16.7%, *p* = 0.67) (Table 2).

## Discussion

Despite a steady rise in numbers of prenatally drug-exposed infants along with an established clinical value and excellent outcomes for neonates receiving ECMO, little is known about the overlay of these two aspects of neonatal intensive care. In this retrospective cohort study, we examined the role that IUDE plays in the treatment and outcomes of neonates requiring ECMO in order to improve sedation and medical management in this vulnerable population. We observed that neonates with and without IUDE did not differ in rates of survival to discharge or the number or type of morbidities. However, neonates with IUDE + ECMO do require more adjuvant therapies for sedation during ECMO. Trending data indicate that neonates with IUDE required greater than 300% higher doses of oral morphine equivalents, may experience more feeding difficulty than those without IUDE, and have a longer length of stay.

Previous studies on neonates who require ECMO show the development of tolerance and the consequent need for increased sedation over the course of their hospital stay (4, 10, 25). This is consistent with our findings that showed all neonates, regardless of the presence of IUDE, required an increase in the amount of sedation and analgesic medication throughout their hospitalization. This was particularly true for neonates with IUDE. The increased sedation requirements for neonates with IUDE is likely due to the increased pain and discomfort experienced secondary to drug withdrawal as well as the development of tolerance to sedative medications *in utero*. In addition, the rapid clearance of maternal drugs from the ECMO circuit may have resulted in earlier and more severe symptoms of withdrawal in neonates with IUDE. Our findings are consistent with studies in adult populations that found the need for higher doses of sedation in patients with previous exposure to opioids or sedative medications (26–28).

Opioid treatment in neonates has been associated with a delay in attainment of full oral feeds (29, 30). This is consistent with our trending results that indicate that neonates with IUDE may take twice as long to reach full oral feeds compared to those without IUDE. The time it takes neonates to reach full oral feeds is a major determinant of length of stay (31, 32). These studies suggest that feeding ability plays a crucial role in determining the length of hospital stay in neonates who require ECMO. Given the role feeding ability plays in length of stay, the delay seen in reaching full oral feeds in neonates with IUDE might explain their increased length of stay compared to neonates without IUDE.

As a result of the findings from this investigation, Kentucky Children's Hospital has developed new clinical practice guidelines (CPG) for the sedation of neonates with IUDE requiring ECMO. These updated guidelines address the increased need for sedative and analgesic medications in neonates with IUDE who are put on ECMO. The CPG include the following: (1) no sedation holidays; (2) use of methadone as the primary medication to control withdraw symptoms; (3) start methadone treatment at 0.3 mg every 12 h, dose can be increased daily by 0.05 mg to a maximum dose of 0.2 mg/kg/dose; (4) consider adding clonidine, phenobarbital, or diazepam as adjuvants therapies; and (5) wean morphine and increase methadone once the neonate is captured. The updated CPG was not used on any neonates in this study. Prospective, multicenter studies should be performed to evaluate the efficacy of the new CPG in controlling the comfort level of neonates with IUDE who require ECMO.

The findings of this study are subject to limitations, which include the small sample size and retrospective study design limited types of analyses we were able to perform. This led to results that were clinically significant but in some cases did not reach the level of statistical significance. Examples of this discrepancy are seen as the number of days it took neonates to reach full oral feeds and the total OME required for pain control and sedation. Additionally, we did not have information on the frequency, timing, or type of drugs that the neonates were exposed to *in utero*. It is possible that these factors impacted the severity of withdraw in the neonates and their response to drugs given in the NICU. For practical reasons, we were also unable to diagnose neonates with IUDE with NAS or capture clinical characteristics of this condition using a standardized scoring system given the critical nature of their illness. We note that our institution is the only level 4 NICU offering ECMO life support to children throughout our region, an area that has been one of the hardest hit from the opiate abuse epidemic (e.g., Central and Eastern Kentucky and Mid-Appalachian US). For these reasons, our patient experiences thus far may be leading other sites, and future studies should include collaborations with other centers to increase the cohort size and to refine and improve clinical guidelines for this unique patient group.

This retrospective study is the first to analyze the impact that IUDE has on the treatment of neonates requiring ECMO life support. We found that neonates with IUDE who require ECMO had no change in survival to discharge or ECMO complications than neonates without IUDE requiring ECMO. However, IUDE was associated with increased need for sedation and analgesic requirements, longer length of hospitalization, and overall more complex care. Our observations suggest that refined strategies and clinical guidelines for this special patient group may be warranted, as well as prospective studies to develop optimized clinical care for improvements in clinical course and outcomes.

## Data Availability

The raw data supporting the conclusions of this article will be made available by the authors, without undue reservation.
